# SGK-Net: A Novel Navigation Scene Graph Generation Network

**DOI:** 10.3390/s24134329

**Published:** 2024-07-03

**Authors:** Wenbin Yang, Hao Qiu, Xiangfeng Luo, Shaorong Xie

**Affiliations:** School of Computer Engineering and Science, Shanghai University, Shanghai 200444, China; youngwb@shu.edu.cn (W.Y.); 1154985402@shu.edu.cn (H.Q.)

**Keywords:** navigation scene graph generation, multimodal fusion, graph structure learning, message passing

## Abstract

Scene graphs can enhance the understanding capability of intelligent ships in navigation scenes. However, the complex entity relationships and the presence of significant noise in contextual information within navigation scenes pose challenges for navigation scene graph generation (NSGG). To address these issues, this paper proposes a novel NSGG network named SGK-Net. This network comprises three innovative modules. The Semantic-Guided Multimodal Fusion (SGMF) module utilizes prior information on relationship semantics to fuse multimodal information and construct relationship features, thereby elucidating the relationships between entities and reducing semantic ambiguity caused by complex relationships. The Graph Structure Learning-based Structure Evolution (GSLSE) module, based on graph structure learning, reduces redundancy in relationship features and optimizes the computational complexity in subsequent contextual message passing. The Key Entity Message Passing (KEMP) module takes full advantage of contextual information to refine relationship features, thereby reducing noise interference from non-key nodes. Furthermore, this paper constructs the first Ship Navigation Scene Graph Simulation dataset, named SNSG-Sim, which provides a foundational dataset for the research on ship navigation SGG. Experimental results on the SNSG-sim dataset demonstrate that our method achieves an improvement of 8.31% (R@50) in the PredCls task and 7.94% (R@50) in the SGCls task compared to the baseline method, validating the effectiveness of our method in navigation scene graph generation.

## 1. Introduction

The development of deep learning-based perception techniques has significantly enhanced the ability of intelligent ships to perceive objects in a scene [1,2]. However, in order to improve the safety of autonomous ship navigation, it is necessary to go beyond surface-level perception and achieve a deeper understanding of the scene. Scene graphs have emerged as an effective way to enhance the scene understanding capability of intelligent ships. However, there are some problems in the current navigation scene image data, such as complex relationships between target entities and noise in the contextual information required for relationship inference. These issues hinder the application of scene graphs in navigation scene understanding. To address these challenges, this paper conducts research on navigation scene graph generation (NSGG).

The scene graph generation (SGG) task is related to scene perception tasks while also demonstrating significant differences. Perception tasks such as object detection [3,4,5], object recognition [6,7,8], and scene segmentation [9,10] primarily focus on acquiring information regarding the category and location of objects. On the other hand, the SGG task emphasizes capturing the relationships between objects within a scene and the interactions between objects and their surrounding environment. Perception tasks primarily rely on computer vision techniques, whereas the task of SGG combines computer vision with natural language processing, making it a higher-level and more complex task [11]. SGG represents a scene using a graph structure, with object detection bounding boxes serving as nodes and the relationships between objects as edges. The generation of scene graphs provides a foundation for subsequent scene understanding tasks and intelligent decision-making. Therefore, in this study, the focus is on generating navigation scene graphs, aiming to enhance the understanding capability of intelligent ships in navigation scenes.

Since the application of scene graphs in image retrieval by Johnson et al. [12], scene graphs have gradually become a research hotspot. Scene graphs model visual information, such as images or videos, and convert them into structured representations that are computationally understandable. They possess the ability to explicitly represent objects, attributes, and relationships, providing support for advanced understanding of complex scenes [13]. Scene graphs can be likened to knowledge graphs in the field of natural language processing [14]. Knowledge graphs contain a wealth of triple information, represented as head entity, relationship, and tail entity. In contrast, scene graphs present abstract entities from natural language processing as concrete image regions. This approach allows for a more intuitive understanding of the relationships between objects in the current scene, but it also adds complexity to the construction of scene graphs.

Currently, research on SGG primarily focuses on indoor scenes [15,16,17], autonomous driving scenes [18,19,20], image retrieval [12,21,22], medical image analysis [23,24], and other fields. For intelligent ships, their navigation scenes are often complex and dynamic. Factors such as varying sea conditions, lighting conditions in navigation environments, and the presence of other dynamically changing ships pose challenges to scene understanding. Scene graphs offer an effective solution to address these challenges. For instance, scene graphs can be used to establish spatial information models of navigation environments, clarifying the positional relationships between static elements such as ships, islands, and ports within the scene. Additionally, scene graphs enable the tracking and modeling of dynamic elements such as other ships, providing insights into their dynamic behavioral attributes and facilitating safe navigation for intelligent ships. In summary, SGG plays a very positive role in understanding navigation scenes for ships, which is also the main motivation for studying the NSGG in this paper.

While the motivation and significance of studying the generation of navigation scene graphs were introduced above, we have encountered a challenging issue in practical research. Most current scene graph research is based on publicly available datasets, which makes it difficult to conduct targeted research on NSGG. Furthermore, the navigation events or object relationships reflected in current real-world navigation image data are limited, which hinders the study of complex navigation scene graph generation. Therefore, it is also not conducive to studying the generation of complex navigation scene graphs. As a result, prior to experiments on NSGG, this paper will also construct a dataset dedicated to NSGG.

In summary, this paper will focus on studying NSGG. Firstly, this paper proposes a novel NSGG network, named SGK-Net, in order to enhance the understanding capability of intelligent ships in navigation scenes. Secondly, this paper also builds the first Ship Navigation Scene Graph Simulation dataset, named SNSG-Sim, for validating the effectiveness of the proposed NSGG method. The main contributions of this paper are as follows:

- To address the challenge of complex and diverse relationships among target entities in the current SGG process, making it difficult to obtain the key relationships that are contextually relevant, we propose the Semantic-Guided Multimodal Fusion (SGMF) module. This module leverages prior information on relationship semantics to fuse multimodal information and construct relationship features, allowing for weighted relationships between entities and providing clarity on the key relationships among target entities in the current context.
- To tackle the issue of redundancy in relationship features during the current SGG process, we propose the Graph Structure Learning-based Structure Evolution (GSLSE) module. This module utilizes graph structure learning to reduce redundancy in relationship features and optimize the computational complexity in subsequent contextual message passing.
- To address the issue of unstable SGG caused by noise interference in the context information relied upon for relational reasoning, this paper proposes the Key Entity Message Passing (KEMP) module. It effectively utilizes context information to refine the relational features and reduce noise interference from non-key nodes.
- In response to the lack of domain-specific datasets for generating navigation scene graphs, this paper introduces the first ship navigation scene graph simulation dataset, SNSG-sim. The dataset consists of 2240 frames of image data captured in different navigation scenes, encompassing 10 common navigation scene entities and 20 inter-entity relationships. This dataset serves as a foundation for research on NSGG.

The subsequent structure of this paper is as follows. Section 2 will provide an overview of related work in the field. Section 3 will introduce the proposed method for generating navigation scene graphs based on semantic-guided multimodal feature fusion and key entity message passing. Section 4 will present the SNSG-sim dataset proposed in this paper. In Section 5, the proposed NSGG method will be compared with existing methods using the SNSG-sim and Visual Genome datasets, followed by an analysis and discussion of the experimental results. Section 6 will summarize the contributions of this paper and provide an outlook on future work.

## 2. Related Work

### 2.1. Scene Graph Generation

The concept of scene graphs was introduced over a decade ago. However, it was not until 2020 that the SGG task gained increasing attention, driven by advancements in pre-training models and datasets such as Visual Genome [25] and Open Images [26]. Current research on SGG can be classified into three categories: 2D SGG, 3D SGG, and spatio-temporal SGG [13]. Among them, there are more datasets available for 2D SGG, and it is a more widely studied area. 2D SGG primarily focuses on objects and their relationships within a single image [27]. RU-Net [28] proposed a regularized unrolling network to alleviate the issue of imbalanced relationship types in training data for generative models. Ref. [29] introduced an SGG method based on causal inference to address biases in the training process of traditional SGG models. Ref. [30] presented a generation method based on region-aware attention learning, which improved the understanding of fine-grained visual regions by SGG models. SGTR [31] and ReITR [32] introduced the transformer to the SGG task, enhancing the relationship modeling capabilities of generative models.

Compared to 2D SGG, Spatio-temporal SGG primarily focuses on the task of SGG in dynamic videos, requiring the consideration of both spatial and temporal dimensions of relationships [33,34,35]. Spatio-temporal scene graphs exhibit stronger expressive capabilities, but they also entail greater challenges in generation and application. With the advancement of perception tasks such as 3D semantic segmentation [9,36,37] and 3D object detection [38,39,40], the 3D SGG task has gained attention in recent years. In contrast to the 2D SGG problem at the image level, understanding and representing the interactions between objects in three-dimensional space typically involve greater complexity [41,42,43]. In terms of scene graph applications, there have been research efforts in various domains such as indoor scenes [15,16,17], autonomous driving scenes [18,19,20], image retrieval [12,21,22], and medical image analysis [23,24]. However, there is currently a scarcity of SGG research specifically focusing on navigation scenes. Therefore, this paper aims to delve into the SGG task in this domain, as it is believed to significantly enhance the understanding of navigation scenes for intelligent ships.

### 2.2. Scene Graph Datasets

Scene graph datasets serve as the foundation for SGG research. Currently, scene graph datasets can be categorized into public datasets and domain-specific datasets. As SGG is a downstream task of object perception, some datasets in the public domain have evolved from object detection or segmentation datasets, such as MSCOCO [44], COCO-Stuff [45], Spatialvoc2k [46], etc. Other datasets are specifically designed for SGG tasks, including VRD [47], Spatialsense [48], Visual Genome [25], Open Images V6 [26], etc. Among them, Visual Genome and Open Images V6 have relatively large numbers of data samples and annotations for objects and relationships, making them the most commonly used public datasets in general SGG research. Diverse public datasets provide an experimental and analytical foundation for SGG research. However, domain-specific SGG research requires datasets specific to the domain. Domain-specific datasets are typically developed in conjunction with specific tasks. For example, refs. [18,19,20] investigated SGG in the context of autonomous driving and proposed datasets for validation. In this paper, we primarily focus on the SGG task in the context of ship navigation scenes. Therefore, we will construct a dataset suitable for training and evaluating the performance of NSGG methods.

## 3. Methodology

### 3.1. Motivation

The generation of scene graphs enables unmanned systems to extract semantic relationships among objects in a scene, thereby facilitating a more comprehensive understanding of the current context. In the specific domain of ship navigation, SGG also contributes to enhancing the scene perception capabilities of intelligent ships. However, the effectiveness of generating navigation scene graphs is hindered by the diversity between the semantic relationships and visual representations of objects in navigation scenes. This manifests as the existence of multiple semantic relationships between the same objects, and significant variations in the visual patterns associated with the same semantic relationship. Consequently, this increases the difficulty of extracting semantic features related to relationships.

**Figure 1:**
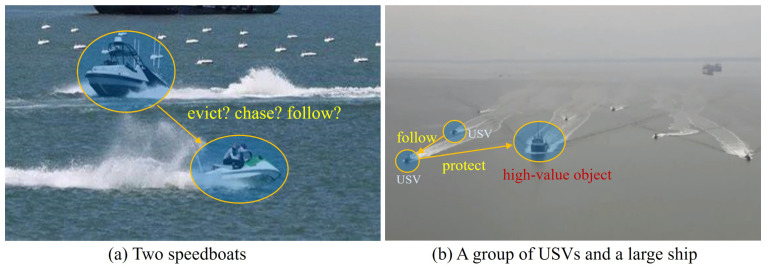
Semantic relationships of objects in different navigation scenes.

As shown in Figure 1a, the visual relationships between the front and rear speedboats can be described by various predicates, such as chase, follow, or evict. The ambiguity of visual relationship semantics makes it difficult to extract specific semantic features. However, in Figure 1b, if the detected intermediate object in the current scene is a high-value object (entity), such as a cargo ship, the contextual information can be used to determine the relationship between the unmanned surface vessels (USVs) and the cargo ship as a protecting relationship, while the relationship between the USVs is likely to be a following relationship. This indicates that relationship inference in the process of generating navigation scene graphs heavily relies on contextual information. Therefore, it is crucial to eliminate noise interference, such as non-key nodes, and identify key objects. To address the diversity of relationships between objects in navigation scenes and the impact of noise interference in contextual information on relationship feature extraction, this paper proposes an NSGG method based on semantic-guided multimodal fusion and key entity message passing, named SGK-Net. The aim of this method is to improve the quality of relationship feature extraction and achieve efficient and high-quality NSGG.

### 3.2. Network Architecture

**Figure 2:**
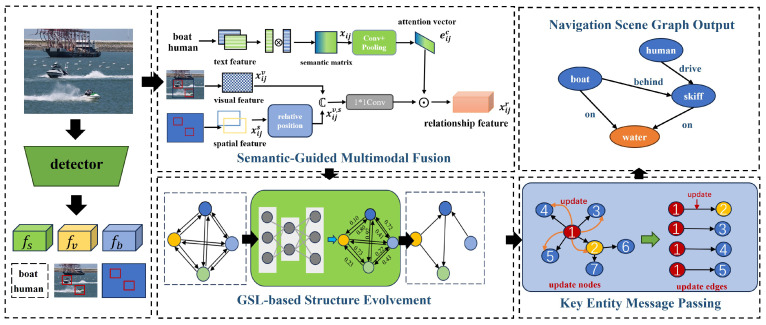
Network Architecture. The SGK-Net comprises three innovative modules: Semantic-Guided Multimodal Fusion (SGMF), Graph Structure Learning-based Structure Evolution (GSLSE), and Key Entity Message Passing (KEMP). These modules aim to address the existing issues in current scene graph generation methods. SGMF is specifically designed to tackle the problem of ambiguous relationships between entities. GSLSE is utilized to optimize the connectivity structure of the navigation scene graph, thereby reducing redundant connections between entity nodes. KEMP is designed to mitigate the interference caused by non-key nodes on relationship inference.

In the research of navigation scene graph generation, challenges arise from the complexity of relationships among navigation objects and the presence of noise in the context information relied upon for relationship inference. To address these challenges, this paper proposes a novel NSGG network, SGK-Net. The overall network architecture of the proposed method is illustrated in Figure 2, and it consists of three innovative modules: the Semantic-Guided Multimodal Fusion (SGMF), the Graph Structure Learning-based Structure Evolvement (GSLSE), and the Key Entity Message Passing (KEMP). Firstly, the SGMF module takes as input the multimodal features and entity information extracted by the detector, and fuses the multimodal features of paired entities to generate relationship features between the entities. This yields a dense representation of relationships. Then, to reduce the propagation of noise information and computational complexity during contextual inference, we utilize the GSLSE module to reconstruct the fully connected graph into a refined relationship graph. Finally, the KEMP module quantifies the weight of each node in the message-passing process based on the connectivity density of the nodes. It relies on the information from important nodes to refine the feature representation of each node in the scene graph, thereby enhancing the accuracy of the nodes and relationships in the scene graph.

### 3.3. Semantic-Guided Multimodal Fusion

Traditional methods often fail to accurately model relationship features, leading to a fragmented understanding of the associations between object multimodal features and overlooking the importance of relationship semantics hidden within the multimodal features. In the context of navigation scenes, the weight of relationship semantics varies across different modalities. For example, spatial features predominantly determine object orientation relationships, while visual information plays a more significant role in object behavioral relationships. Neglecting the prior semantic clues between objects can result in ambiguous relationship semantics, thereby affecting the generation of navigation scene graphs. To address this issue, this paper proposes the Semantic-Guided Multimodal Fusion module (SGMF), which focuses on the prior relationships between object categories. It leverages this prior information to enhance the visual and spatial feature representations between object pairs, thereby strengthening the representation of multimodal relationship features and mitigating the problem of semantic ambiguity in object relationships caused by missing information in multi-modal relationship features.

First, the image is processed using an object detector to obtain the semantic label information $f_{s}$, visual information $f_{v}$, and bounding box information $f_{b}$ of the objects in the image. In previous works, $f_{v}$ is commonly represented using a joint region representation. Typically, the multi-modal information contained in the bounding boxes of two objects is used to represent the relational features. The features from different modalities are concatenated to form a fused feature, where different channels focus on different patterns of relational information. However, the resulting relational features inevitably get mixed with a large amount of unrelated background information. The SGMF module is designed to extract the relevant patterns of relationship features by leveraging the prior semantic association information between paired objects. Specifically, given a subject *i* and an object *j*, the semantic feature vectors obtained from the detector are denoted as $f_{se}\left(c_{i}\right)$ and $f_{se}\left(c_{j}\right)$, respectively. The semantic prior matrix $x_{ij}$, representing the semantic correlation between the object pair, is computed as:

(1) $$x_{ij}=f_{se}\left(c_{i}\right)⊗f_{se}{\left(c_{j}\right)}^{T}.$$

The symbol ⊗ denotes matrix multiplication. Next, we compute the spatial relationship features between the object pairs based on the object detection bounding boxes obtained from the detector. In this paper, the calculation formula for the spatial relationship feature $x_{ij}^{s}$ is as follows:

(2) $$x_{ij}^{s}=\sigma \left(\frac{x_{i}-x_{j}}{w_{j}},\frac{y_{i}-y_{j}}{h_{j}},\log\frac{w_{i}}{w_{j}},\log\frac{h_{i}}{h_{j}}\right),$$

where $\left(x_{i},y_{i},w_{i},h_{i}\right)\in b_{i}$, $\left(x_{j},y_{j},w_{j},h_{j}\right)\in b_{j}$ represents the spatial prior information between the objects. As shown in Figure 2, the SGMF module then performs a channel-level fusion of the visual relationship representation $x_{ij}^{v}$ and the spatial relationship feature $x_{ij}^{s}$. Therefore, the multimodal relationship feature $x_{ij}^{v,s}$, which captures both visual and spatial information, can be represented as:

(3) $$x_{ij}^{v,s}=cat\left(x_{ij}^{v},x_{ij}^{s}\right).$$

Next, a series of 2D convolutions and spatial pooling on $x_{ij}$ are performed to achieve channel attention, resulting in the attention vector $e_{ij}^{c}$. The obtained semantic relationship attention vector is then combined with the multi-modal relationship features through channel-wise operations to update the feature representation of the relationship. This process helps select feature patterns that have stronger semantic correlations.

(4) $$e_{ij}^{c}=\sigma (G_{pooling}\left(G_{conv}^{n}\ldots \sigma \left(G_{conv}^{1}\left(x_{ij}\right)\right)\right)$$

(5) $$x_{ij}^{r}=x_{ij}^{v,s}\times e_{ij}^{c},$$

where *c* is the number of channels in the multi-modal relationship feature $x_{ij}^{v,s}$, σ represents the activation function, $G_{conv}$ denotes the 2D convolution operation, $G_{pooling}$ represents the 2D pooling operation. Finally, $x_{ij}^{r}$ is obtained as the updated relationship feature, with × indicating the dot product.

### 3.4. Graph Structure Learning-Based Structure Evolvement

In previous research, to improve the prediction accuracy of relationships in scene graphs, it is common to model the relationships between each pair of objects. If *N* objects are detected in an image, the constructed scene graph would involve $N^{2}$ relationship features that need to be processed and predicted. For instance, MOTIFS [49] takes the features of all objects and feeds them into a Bi-LSTM to generate $N^{2}$ relationship features. MSDN [50] groups N objects into N(N−1) pairs, excluding pairs with identical objects, and connects all different objects using directed edges. However, such approaches have negative impacts on learning contextual features in the following ways: (1) Noise data are propagated during the context message passing process. Some noisy nodes transmit their information in this dense graph, significantly affecting the convergence of features in other parts of the graph; (2) The computational complexity of dense graph connections is substantial, making it challenging for the entire model to converge. To address these issues, this paper draws inspiration from graph learning theory and proposes the graph structure learning-based structure evolvement module to simplify dense scene graphs and select appropriate connections as much as possible.

In the GSLSE module, the relationship feature $x_{ij}^{r}$ is first fed into the GSLSE module to generate the edge-weighted skeleton graph or structured organization rule set $S_{G}=\left\{r_{1,2},r_{2,3},\ldots ,r_{n,n-1}\right\}$. The GSLSE module can be regarded as a binary classification task to determine the existence of associations between nodes, and its confidence reflects the degree of association between nodes in the scene graph. By replacing the edges with classification confidences, the fully connected graph is reconstructed into an edge-weighted skeleton graph. The implemented GSLSE module in this section consists of three fully connected layers, with ReLU non-linear activation functions applied between each layer. GSLSE directly takes the initial relationship feature $x_{ij}^{r}$ as input and predicts the association between the objects $o_{i}$ and $o_{j}$. GSLSE only considers the positive values of the classification results as the final confidence scores, which are then normalized using softmax. To control the high or low confidence scores, these scores are input into a gating function τ(·) proposed by [51], which helps align the predicted organization closer to the skeleton of the ground truth scene graph. The entire process is illustrated as follows:

(6) $$r_{i,j}=\tau (softmax\left(GSLSE\left(x_{ij}^{r}\right)\right)$$

(7) $$\tau \left(x\right)=\left\{\begin{array}{cc} 0 & x\leq \beta \\ \alpha x-\alpha \beta & \beta <x<\frac{1}{\alpha }+\beta \\ 1 & x\geq \frac{1}{\alpha }+\beta \end{array}\right.,$$

where α and β are two learnable parameters. The function τ(·) is used to process the result of the softmax function.

After the processing by the SGMF module in the previous section, a dense connectivity graph of the entire scene is obtained. The GSLSE module significantly reduces the connectivity between nodes in the graph. The GSLSE module’s processing not only reduces the propagation of redundant connection noise but also decreases the computational complexity during scene graph inference.

### 3.5. Key Entity Message Passing

Through the analysis conducted in Section 3.1, it has been observed that the improvement in the effectiveness of NSGG relies heavily on contextual information. Information is primarily conveyed through messages, wherein messages represent the essence of features and are exchanged between elements of the scene graph, including objects and relationships. For the source elements, messages are considered as their own features, while for the target elements, they serve as contextual information. Intuitively, predictions regarding objects and relationships can benefit from their contextual information. For instance, in the IMP [52] method, separate feature representations are constructed for nodes and relationships. Dual-form GRUs are utilized to transmit contextual information between nodes and edge contexts, facilitating information propagation between nodes and edges. VCTREE [53], on the other hand, employs a dynamic tree structure to place objects within visual contexts and utilizes bidirectional TreeLSTM to encode the visual contexts. However, the current utilization of contextual mechanisms suffers from a problem where the weight of entity information transmission remains consistent during the message-passing process. This uniform weight distribution leads to the overlooking of important entity information and the propagation of more noise information when understanding the contextual cues of navigation scenes. To address this issue, this section proposes the Key Entity Message Passing (KEMP) module to optimize the contextual propagation process.

Specifically, the first step involves utilizing the graph skeleton structure $S_{G}$ generated by the GSLSE module, which aggregates the connectivity information of all nodes in the graph. The connectivity status of the graph forms the basis for selecting important entities in the scene. The degree of each node can be calculated based on its connectivity status. We define the importance of an entity to be positively correlated with the degree of its corresponding node, thus quantifying the importance of entities. The calculation of the degree of a node $o_{i}$ is given by the following formula:

(8) $$d_{o_{i}}=\sum_{j\in N_{*i}}^{\;}\left(r_{i,j}*I\left(r_{i,j}\right)\right),$$

where the function I(·) is an indicator function. It takes a condition $r_{i,j}$ as input and returns a value of 1 if $r_{i,j}$ is true and 0 if $r_{i,j}$ is false. Subsequently, in the message-passing process, to highlight the importance of neighboring nodes in aggregating neighborhood information for node $o_{i}$, we employ a weight-based message aggregation approach. The context weights are calculated based on the degrees of neighboring nodes as follows:

(9) $$Z_{d_{o_{i}}}=\frac{d_{o_{i}}-min\left(d_{o}\right)}{max(d_{o})},$$

where $d_{o}$ represents the set of node degrees, and Z(·) is the weight function for the node degrees. Next, in the message passing process, to highlight the importance of neighboring nodes in aggregating information within the neighborhood of node $o_{i}$, we employ a weight-based message aggregation method. The context weights are calculated based on the out-degree of neighboring nodes, and the calculation is formalized as Z(·). Subsequently, we use $o_{i}$ and $x_{ij}^{r}$ to initialize the hidden state: $E_{o_{i}}^{\left(0\right)}=o_{i}$, $V_{r_{ij}}^{\left(0\right)}=x_{ij}^{r}$. The formal expression for context message passing of key entities is as follows:

(10) $$\begin{array}{cc} E_{o_{i}}^{\left(l\right)} & =g^{e\to e}\left(r_{i,j},d_{o_{j}},E_{o_{j}}^{\left(l-1\right)}\right) \end{array}$$

(11) $$\begin{array}{cc} \; & =\sum_{j\in N_{*i}}\left(r_{i,j}\cdot Z_{d_{o_{i}}}\cdot E_{o_{i}}^{\left(l-1\right)}+E_{o_{i}}^{\left(l-1\right)}\right) \end{array}$$

(12) $$\begin{array}{cc} V_{r_{ij}}^{\left(l\right)} & =g^{e\to r}\left(r_{i,j},E_{o_{i}}^{\left(l-1\right)},E_{o_{j}}^{\left(l-1\right)}\right) \end{array}$$

(13) $$\begin{array}{cc} \; & =\lambda \left[g^{s}\left(r_{i,j}\cdot E_{o_{i}}^{\left(l-1\right)}\right)+g^{o}\left(r_{i,j}\cdot E_{o_{j}}^{\left(l-1\right)}\right)\right]+V_{r_{ij}}^{\left(l-1\right)}, \end{array}$$

where $E_{o_{i}}^{\left(l\right)}$ represents the node features after l-th layer message passing, $V_{r_{ij}}^{\left(l\right)}$ represents the relationship features after l-th layer message passing, $N_{*i}$ denotes the neighborhood of node $o_{i}$. e→r signifies feature propagation between nodes, while e→r denotes the utilization of node information to update edge features. $g^{e\to e}$, $g^{e\to r}$, $g^{s}$, $g^{o}$, etc., all refer to multi-layer perceptrons used to refine hidden layer representations. λ represents a learnable value.

### 3.6. Training Losses

The model training in this study is performed using supervised learning. The overall loss of the NSGG model, denoted as $L_{all}$, is divided into three components: graph structure training loss $L_{gslse}$, object detection loss $L_{obj}$, and relation predicate prediction loss $L_{rel}$:

(14) $$L_{all}=L_{obj}+L_{rel}+L_{gslse}.$$

The $L_{obj}$ component is primarily used to determine the object class and object region in the image. The $L_{obj}$ loss is composed of classification loss and regression loss. In this study, the classification loss is calculated using cross-entropy loss, while the regression loss is calculated using smooth L1 loss. On the other hand, $L_{rel}$ is mainly used to learn the relationships between objects, and this study employs cross-entropy loss for this purpose. Now, let us focus on introducing the graph structure training loss $L_{gslse}$, employed in the GSLSE module in this study.

The traditional SGG models are typically supervised with triplets in the form of $<e_{i},e_{ij},e_{j}>$, rather than the intuitive representation of scene graphs. This representation lacks structural and hierarchical information, resulting in a deficiency of explicit relationships. As a consequence, the translation of visually structured information into implicit representations leads to suboptimal performance. To address this issue, we propose the GSLSE module to generate the graph structure $S_{G}$, while the ground truth structure representation of the scene graph is denoted as:

(15) $$S_{GT}=\left\{b_{12},b_{13},\ldots ,bn_{n-1}\right\},$$

where $b_{ij}=1$ indicates the existence of an edge from $o_{i}$ to $o_{j}$, and when $b_{ij}=0$, the edge does not exist. The loss of the GSLSE module is then defined as follows:

(16) $$L_{gslse}=BCE\left(S_{G},S_{GT}\right).$$

The binary cross-entropy loss function, BCE(·), is utilized in this context. It is worth noting that our graph structure data does not include any labels for nodes and edges; it solely contains information that constrains the model to learn structured graph representations.

By jointly training multiple tasks, we can prevent modules from converging independently in a static space, thus effectively integrating structured information into feature learning.

## 4. SNSG-Sim Dataset

Due to the limited availability of object relationships in existing ship navigation scene image data, and the scarcity of data suitable for scene graph generation tasks, there is currently no publicly available dataset specifically designed for ship navigation scene graph generation research. To facilitate a better understanding of ship navigation scene graph generation, this study presents the first Ship Navigation Scene Graph Simulation dataset, SNSG-sim. This dataset is constructed utilizing the Unity3D engine, taking into consideration various object relationships in different navigation scenes. It comprises a total of 2240 image data samples from diverse navigation scenes such as islands, ports, and open seas. The dataset includes 10 types of navigational entities (objects), including cargo ships, cruise ships, and USVs, as well as 20 common navigational object relationships.

### 4.1. Dataset Construction

To construct a simulation scene graph dataset, it is crucial to have a stable simulation platform that can accurately simulate the objects and backgrounds encountered during ship navigation. Existing scene graph generation datasets primarily rely on data collection through networks, lacking targeted domain-specific data and lacking precedents for constructing navigation scene graph datasets based on simulation environments. Therefore, in this study, the selection of the simulation environment was guided by the upstream perception task datasets, such as Vkitti [54] and S2S-sim [1], which utilized Unity3D as the simulation platform. Building upon this simulation engine, the scenes were constructed for the dataset.

**Figure 3:**
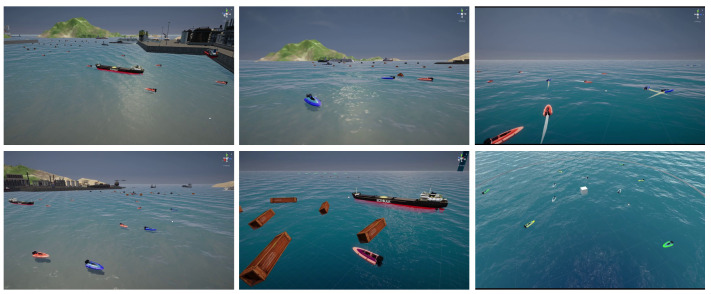
Simulation of common navigation scenes.

**Figure 4:**
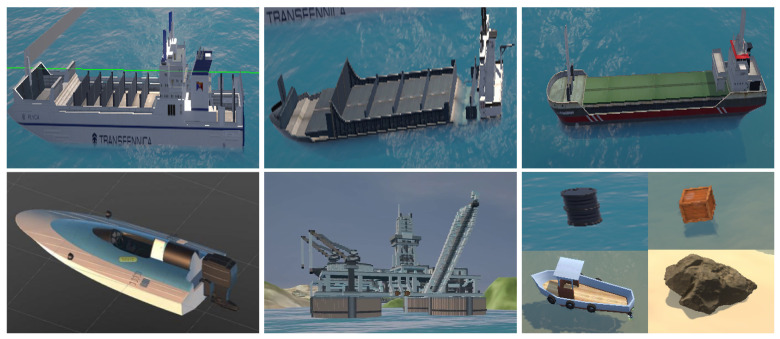
Part of objects appearing in simulated navigation scenes.

**Figure 5:**
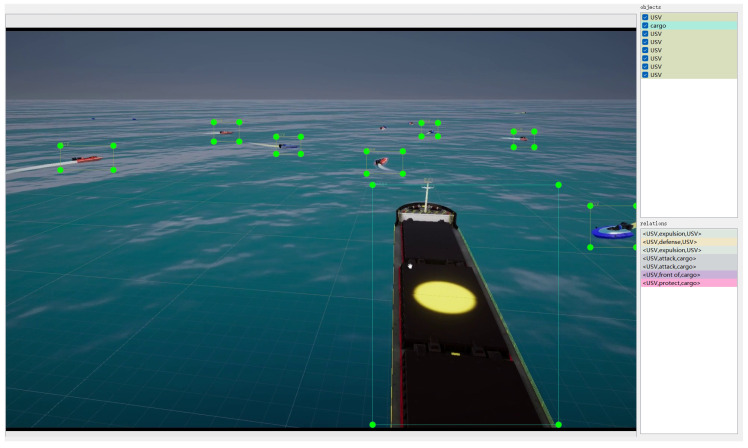
An example of entity and relationship annotation for attack and defense entities is shown in the figure. The cargo ship is the main object, while the defense USV is depicted in red, and the attack USV is depicted in blue.

In the construction of scenes, this study refers to commonly observed navigation scenes and primarily divides them into port, island, and open sea, as shown in Figure 3. Regarding the selection of target entities, in addition to common cargo ships, cruise ships, and USVs, it also includes offshore drilling platforms (ODPs) and other obstacles, as illustrated in Figure 4. In terms of entity relationship selection, apart from intuitive relationships such as “follow” and “beside”, we have also identified more complex implicit relationships, such as “defense” and “rescue”, which require contextual understanding through simulation. The specific entity and relationship annotations are presented in Figure 5. The object labels we annotated include categories and bounding boxes, while relationships are stored in the form of triplets, such as <USV, defense, cargo>. Based on simulations and collected actual samples, we eventually selected and annotated a total of 2240 image data samples, encompassing 20 types of entity relationships.

### 4.2. Dataset Analysis

**Figure 6:**
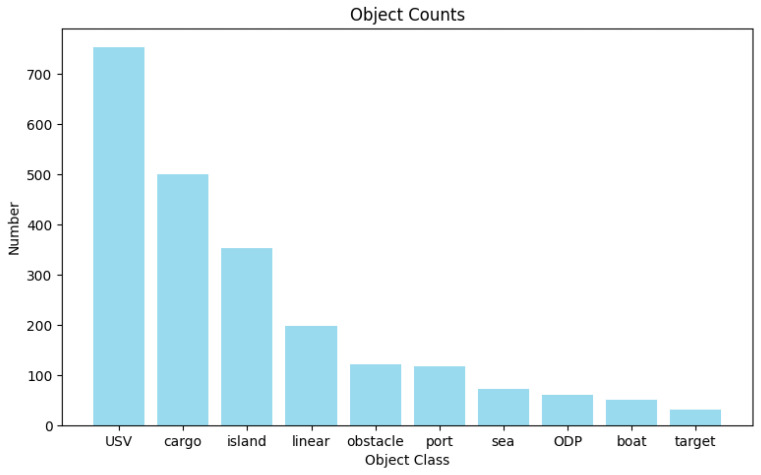
Distribution of entities (objects) in the SNSG-sim dataset.

**Figure 7:**
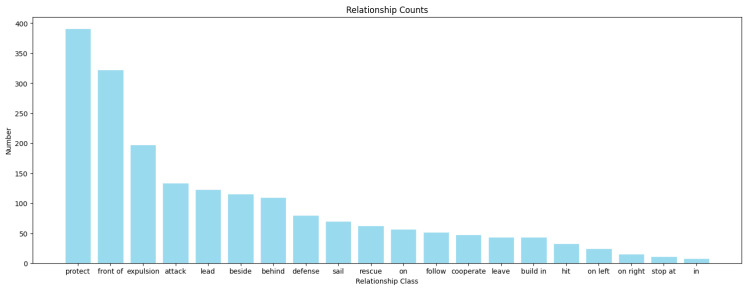
Distribution of relationship in the SNSG-sim dataset.

We conducted a statistical analysis of the constructed SNSG-sim dataset. Figure 6 illustrates the distribution of target entities in the dataset. It can be observed that the annotated target entities in our dataset include foreground objects such as USVs, cargo ships, and cruise ships, as well as background elements like ports and islands, commonly found in navigation scenes. Among them, USVs have a relatively higher representation. This is because, in our dataset, USVs tend to appear in groups during tasks such as protection and defense, leading to a higher frequency of occurrence within the same image sample. Figure 7 displays the distribution of relationships in the constructed dataset. As mentioned earlier, our dataset specifically annotates 20 types of relationships between target entities, addressing the characteristics of navigation scenes. This augmentation enhances the diversity of scene graph generation in our study and improves the understanding of various navigation scenes by using ship models. Furthermore, the statistical graph reveals a similar long-tail distribution issue in both target entities and relationships within navigation scenes, as observed in public scene graph datasets [25,26]. This poses a challenge to the performance of NSGG methods.

## 5. Experiments

In this section, we will evaluate and analyze the proposed NSGG method through experiments on the SNSG-sim dataset. Additionally, we will also conduct experiments on the widely used public dataset, Visual Genome, to validate the generalizability of the proposed method.

### 5.1. Scene Graph Evaluation Tasks and Metrics

In order to better evaluate the learning and reasoning capabilities of the scene graph generation model regarding relationship predicates, this study selects two commonly used evaluation tasks in scene graph generation:

**Predicate Classification (PredCls):** The PredCls task involves predicting the predicates that describe the relationships between pairs of localized objects. This task independently examines the model’s performance in terms of predicate classification without considering other factors. It is primarily used to assess the model’s ability to learn predicates.

**Scene Graph Classification (SGCls):** The SGCls task involves predicting the object categories and predicates for each subject-object pair within a given set of localized objects. This task is primarily used to evaluate the scene graph generation model’s ability to perform contextual reasoning.

Common evaluation metrics used in SGG include Recall@K and mean Recall@K. Recall@K measures the proportion of correctly predicted relationships among the top K-predicted relationships when sorted by confidence scores. In our experiments on the SNSG-sim dataset, we set K values to 20 and 50. A higher value of this metric indicates better predictive performance of the model. The basic formula for recall R@K is as follows:

(17) $$R@K=\frac{1}{Y}\sum_{y=1}^{Y}\frac{T_{y}^{k}}{G_{y}},$$

where *Y* represents the number of images in the test set. After the model’s computation, *K* relationship triplets will be generated for each image. $G_{y}$ represents the number of annotated relationship triplets in the dataset used, and $T_{y}^{k}$ represents the number of predicted values that match the ground truth.

The mean Recall@K is also an important metric for evaluating SGG. The formula for calculating mean recall R@K is as follows:

(18) $$mR@K=\frac{1}{X}\sum_{x=1}^{X}R@K_{x},$$

where *X* represents the number of relationships in the test set. The recall $R@K_{x}$ for each relationship category is computed, and the mean recall mR@K can be obtained by taking the mean of these recall values.

### 5.2. Experimental Settings

**Dataset Settings:** In the experimental section, the experiments mainly utilize the proposed SNSG-sim dataset. For the experiments, we partitioned the 2240 data samples into training and testing sets in a 4:1 ratio. Additionally, to evaluate the generalizability of our proposed method, we also conducted experiments on the publicly available Visual Genome dataset. The Visual Genome dataset consists of over 100,000 images, with the testing set containing 5000 sample images. On average, each image in the dataset is annotated with 35 object labels, 26 attribute labels, and 21 relationship labels. The dataset covers a wide range of everyday life scenes and complex social scenes. Due to the larger number of objects and attributes in the Visual Genome dataset, we followed the settings of other methods and set the value of K (Recall@K) to 50 and 100 during testing.

**Implementation Details:** We adopted the currently prevalent framework for scene graph generation implementation. The scene graph generation model was trained by incorporating a relationship prediction head into the Mask R-CNN algorithm framework. In our approach, the object detection component of the scene graph generation network utilized Faster R-CNN as the detector, with ResNet-101 serving as the backbone network, and the output dimension of the backbone network was set to 256. During training, we first pre-trained the Faster R-CNN object detector. Subsequently, when training the scene graph model, we froze all parameters before the ROIAlign network in Faster R-CNN. For each image, only the top 64 object candidates from the output of Faster R-CNN were retained. During the training process, a batch size of 12 was used, and the learning rate was set to 0.0015. The optimization was performed using the SGD (Stochastic Gradient Descent) algorithm, with a momentum factor of 0.81. The maximum number of iterations for the training process was set to 50,000. All experiments were conducted using one RTX 4090 GPU.

### 5.3. Quantitative Results and Comparison

**Figure 8:**
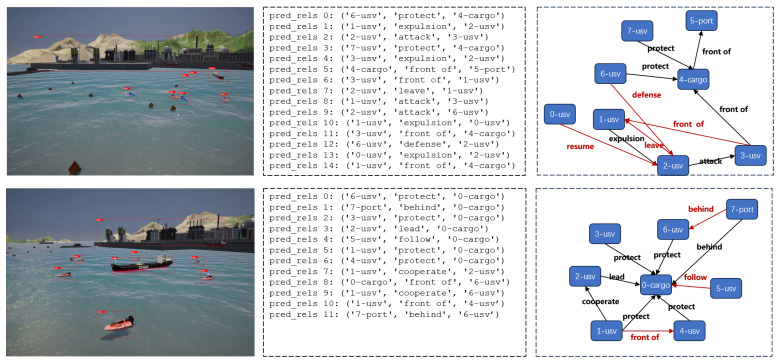
The visualization results of our method on the SNSG-sim dataset for scene graph generation.

**SNSG-sim test results.** The comparative experimental results on the SNSG-sim dataset are presented in Table 1. From the table, it can be observed that our proposed NSGG method exhibits superior performance in both the PreCLs and SGCls evaluation tasks. Thanks to the targeted design of our method for navigation scenes, it is the only method in the PreCLs task that achieves a recall rate of over 60% at both R@20 and R@50. Compared to the baseline method RU-Net, our method achieves improvements of 9.1% and 8.3% in R@20 and R@50, respectively, for the PreCLs task, and improvements of 7.7% and 8.0% in R@20 and R@50, respectively, for the SGCls task. Compared to the recent transformer-based work RelTR [32], our method exhibits significant improvements of 6.9% and 6.0% in R@20 and R@50, respectively, for the PreCLs task, and remarkable improvements of 15.2% and 13.9% in R@20 and R@50, respectively, for the SGCls task. These results validate the effectiveness of our method in generating navigation scene graphs. Figure 8 presents the visualization of our method for generating scene graphs on sample images from the SNSG-sim dataset. It can be observed that the generated scene graphs by our method accurately depict the objects and relationships in navigation scenes.

**Figure 9:**
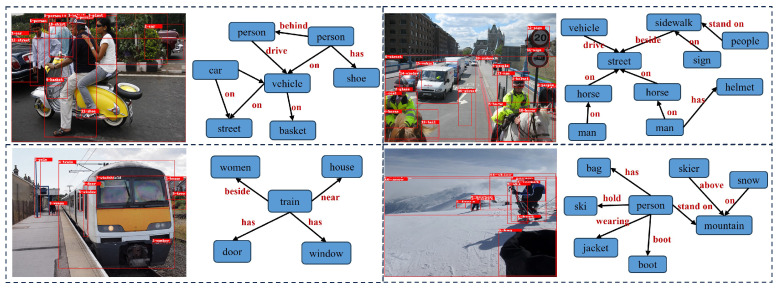
The visualization results of our method on the Visual Genome dataset for scene graph generation.

**Visual Genome test results.** The comparative experimental results on the Visual Genome dataset are presented in Table 2. Since most existing methods have been evaluated on the Visual Genome dataset, we are able to compare our method with a larger number of approaches on this dataset. It can be observed that the proposed scene graph generation method in this paper can also adapt to large-scale visual understanding tasks and generate high-quality scene graphs. Compared to current mainstream methods, our method achieves the best performance in both evaluation tasks. Specifically, the proposed method achieves an R@50 of 69.1% and an R@100 of 71.1% for the PreCLs task on the Visual Genome dataset. For the SGCls task, the method achieves an R@50 of 42.7% and an R@100 of 43.9%. Compared to the baseline model RU-Net, our method improves the R@50 by 1.4% and 1.5% for the PreCLs task and by 1.3% and 1.4% for the SGCls task. Compared to the recent work of RelTR, our method also shows significant improvements in the R@50 metric, with an increase of 4.9% for the PreCLs task and 6.1% for the SGCls task. Figure 9 showcases the visualization of our method for generating scene graphs on sample images from the Visual Genome dataset. These experimental results demonstrate the effectiveness of the proposed method in generating scene graphs on the Visual Genome dataset, thus confirming the generalizability of our method.

### 5.4. Ablation Studies

In order to validate the effectiveness of the innovative modules in our proposed NSGG method, we conducted ablation experiments on both the SNSG-sim dataset and the Visual Genome dataset. In the ablation experiments, the baseline method used was RU-Net, and the recall rate K values for all experiments were set to 20 and 50.

Table 3 presents the ablation experiment results of our method on the SNSG-sim dataset. It can be observed that, compared with the baseline EXP.1, the EXP.2 and EXP.3 that introduced SGMF or GSLSE modules achieve improved performance on both PreCls and SGCls evaluation tasks, and the performance gain brought by the GSLSE module is particularly significant. However, in the EXP.4, which solely introduced the KEMP module, the model performance did not show a significant improvement compared with the baseline method. But in EXP.5, combining GSLSE and KEMP further improved the performance. The main reason is that GSLSE optimizes the scene graph structure, making the message passing between key nodes in the KEMP module more smooth. In EXP.6, introducing all three modules proposed in this paper achieved the best performance, indicating that combining the use of all modules designed in this paper is the most reasonable approach.

Table 4 presents the ablation experiment results of our method on the Visual Genome dataset. From EXP.2, it can be observed that compared to the baseline method, the introduction of the SGMF module leads to a 1.3% improvement in R@50 for the PreCls task. This indicates that the SGMF module is capable of better modeling relationship features. From EXP.3, it can be seen that the introduction of the GSLSE module results in a 1.8% increase in R@50 for the SGCls task. This demonstrates that optimizing the scene graph’s connectivity through the GSLSE effectively facilitates feature learning. Similar to the ablation experiments conducted on the SNSG-sim dataset, the introduction of the KEMP module in EXP.4 does not show significant improvements. However, by analyzing EXP.4 and EXP.5 together, it can be observed that the KEMP module requires the assistance of the GSLSE module to unleash its effectiveness. This relates to the computation of key nodes, where GSLSE aids the KEMP module in calculating key nodes within the scene graph. EXP.6 further demonstrates that the combined usage of the proposed modules in this paper achieves the best performance in both the PreCls and SGCls evaluation tasks. This validates the effectiveness of the innovative modules proposed in this paper.

## 6. Conclusions and Future Work

To address the challenges of generating navigation scene graphs with poor performance due to the complexity of relationships between objects and the presence of noise in the contextual information relied upon for relationship inference, this paper proposes a novel NSGG network, SGK-Net. The proposed network comprises three innovative modules: SGMF, GSLSE, and KEMP. (1) The SGMF module leverages prior information about relationship semantics to fuse multimodal information and construct relationship features, reducing the semantic ambiguity of relationships between objects caused by missing multimodal relationship feature information. (2) The GSLSE module reduces redundancy in relationship features and optimizes subsequent message propagation calculations. (3) The KEMP module utilizes contextual information to refine relationship features and mitigate noise interference from non-key nodes. Additionally, (4) this paper introduces the SNSG-sim dataset, the first simulated dataset for ship navigation scene graph generation, providing a valuable data foundation for research in this area. The effectiveness and generalizability of the proposed method are validated through experiments conducted on the SNSG-sim dataset and the publicly available Visual Genome dataset. Ablation experiments demonstrate that the coordinated operation of the innovative modules in this paper enables the effective extraction of relationship features between target entities, resulting in the generation of navigation scene graphs.

This paper focuses on the NSGG task, and the proposed method provides valuable insights into improving the understanding of navigation scenes for intelligent ships. However, there are also some limitations of this research, primarily in two respects. Firstly, although the SGMF module in this paper achieves a fusion of multimodal features, there still exists a semantic gap between visual representation and relationship predicates, which hinders the effectiveness of scene graphs. In future work, we will pay more attention to addressing the semantic representation issues across different modalities. Secondly, the research in this paper is primarily based on the simulated dataset SNSG-sim, without conducting experiments in real navigation scenes. However, there exist domain differences between real-world data and simulated data. Therefore, transferring the proposed method to real navigation scenes to enhance its practicality is a task we need to accomplish in the future.

## Figures and Tables

**Table 1:** Performance comparisons with state-of-the-art methods on SNSG-sim dataset.

Method	B		SGCls	
	**R@20**	**R@50**	B	**R@50**
MOTIFS [49]	54.4	55.9	40.5	41.6
IMP [52]	48.6	49.3	42.3	44.1
VCTREE [53]	59.6	60.7	29.4	31.7
HL-Net [27]	57.2	59.1	51.9	52.7
RU-Net [28]	52.9	54.0	49.5	50.3
RelTR [32]	55.1	56.3	42.1	44.4
Ours	**62.0**	**62.3**	**57.3**	**58.3**

**Table 2:** Performance comparisons with state-of-the-art methods on the Visual Genome dataset.

Method	PreCls		SGCls	
	**R@50**	**R@100**	**R@50**	**R@100**
MOTIFS [49]	65.8	67.1	35.8	36.5
IMP [52]	59.3	61.3	34.6	35.7
GPI [55]	65.1	66.9	36.5	38.8
VCTREE [53]	66.4	68.1	38.1	38.8
GPS-Net [56]	66.9	68.8	39.2	40.1
G-RCNN [57]	54.2	59.1	29.6	31.6
SGGNLS [58]	65.6	67.4	40.0	40.8
Seq2Seq-RL [59]	66.4	68.5	38.3	39.0
RU-Net [28]	67.7	69.6	41.4	42.3
RelTR [32]	64.2	-	36.6	-
Ours	**69.1**	**71.1**	**42.7**	**43.6**

**Table 3:** Ablation experiments on SNSG-sim dataset.

EXP.	SGMF	GSLSE	KEMP	PreCls		SGCls	
				**R@20**	**R@50**	**R@20**	**R@50**
1				52.8	54.0	49.5	50.3
2	✓			54.2	55.7	52.2	53.5
3		✓		57.8	58.3	54.6	55.2
4			✓	52.3	55.3	48.3	50.1
5		✓	✓	60.6	61.1	55.7	56.4
6	✓	✓	✓	62.0	62.3	57.3	58.3

**Table 4:** Ablation experiments on the Visual Genome dataset.

EXP.	SGMF	GSLSE	KEMP	PreCls		SGCls	
				**R@20**	**R@50**	**R@20**	**R@50**
1				57.6	63.4	35.2	35.3
2	✓			58.2	64.7	35.8	35.9
3		✓		59.3	66.3	36.2	37.1
4			✓	58.1	65.0	35.0	37.4
5		✓	✓	61.7	68.3	37.4	42.5
6	✓	✓	✓	62.3	69.1	42.7	43.6

## Data Availability

The data required to reproduce these findings cannot be shared at this time as the data also form part of an ongoing study. They can be requested from the author by e-mail (youngwb@shu.edu.cn) in the future.
